# Mechanism Analysis for the Enhancement of Low-Temperature Impact Toughness of Nodular Cast Iron by Heat Treatment

**DOI:** 10.3390/ma17020513

**Published:** 2024-01-21

**Authors:** Huanyu Zhuang, Jiahui Shen, Minhua Yu, Xulong An, Jing Hu

**Affiliations:** 1 Jiangsu Key Laboratory of Materials Surface Science and Technology, National Experimental Demonstration Center for Materials Science and Engineering, Changzhou University, Changzhou 213164, China; 13815053984@163.com (H.Z.); 15539719973@163.com (J.S.); axl@cczu.edu.cn (X.A.); 2 Huaide College, Changzhou University, Jingjiang 214500, China; 3 Integrated Management Department, Jiangsu Shuangliang Boiler Co., Ltd., Jiangyin 214444, China; 15861193706@163.com

**Keywords:** nodular cast iron, impact toughness, heat treatment, mechanism, high-angle grain boundaries (HAGB)

## Abstract

The low-temperature impact toughness of nodular cast iron can be significantly enhanced by heat treatment, and thus meet the severe service requirements in the fields of high-speed rail and power generation, etc. In order to explore the enhancement mechanism, microstructure, hardness, composition and other characteristics of as-cast and heat-treated nodular cast iron is systematically tested and compared by optical microscopy, microhardness tester, EBSD, SEM, electron probe, and impact toughness testing machine in this study. The results show that heat treatment has little effect on the morphology and size of graphite in nodular cast iron, ignores the effect on the grain size, morphology, and distribution of ferritic matrix, and has little effect on the hardness and exchange of elements, while it is meaningful to find that heat treatment brings about significant decrease in high-angle grain boundaries (HAGB) between 59° and 60°, decreasing from 10% to 3%. Therefore, the significant enhancement of low-temperature impact toughness of nodular cast iron by heat treatment may result from the obvious decrease in HAGB between 59° and 60°, instead of other reasons. From this perspective, the study can provide novel ideas for optimizing the heat treatment process of nodular cast iron.

## 1. Introduction

Cast iron, a versatile material utilized extensively in the engineering field, has obtained global recognition and application due to its unique combination of castability, machinability, and a relatively low manufacturing cost [1,2,3]. Its characteristics, including high melting point and excellent fluidity make it a prime choice in various engineering applications. Among them, one specific type of cast iron, known as nodular cast iron, has emerged as an even more advantageous material in certain applications due to its distinct characteristic—the presence of graphite in spherical form [4,5,6].

Nodular cast iron, due to its unique microstructural attribution, makes it distinguished within the cast iron family. Unlike the flaky graphite structure existed in grey cast iron, graphite exists in the form of tiny spheres in nodular cast iron. This spherical shape of graphite minimizes the stress concentration and distributes more uniformly in the matrix, thereby bringing about significant advantages of owning good strength and toughness as well. Furthermore, it exhibits excellent wear resistance and seismic resistance, a testament to its versatility.

The significant advantages of nodular cast iron have led to its wide applications across various engineering fields. In railway and subway components manufacturing, its wear resistance and seismic resistance are critical attributes that make it an ideal choice. Similarly, in the production of locomotive and vehicle components, nodular cast iron is preferred due to its high strength, hardness, and toughness. In the petroleum and petrochemical equipment industry, nodular cast iron is favored due to its mechanical resilience and durability. Even in sectors like wind turbine hub and car gearbox manufacturing, ductile iron is recognized and utilized due to its excellent performance characteristics coupled with cost-effectiveness. 

However, like all kinds of materials, ductile iron also has limitations. A significant challenge is its poor performance in deep low-temperature environments; its poor impact toughness renders it liable to result in brittle fracture under severe conditions [7,8,9].

To overcome this disadvantage, heat treatment has been adopted and proven to be an effective technology. Heat treatment is a kind of technology by conducting heating and cooling process to adjust the microstructure, and thus improve the needed mechanical properties and enhance the service life of components in engineering applications [9,10,11,12,13]. For nodular cast iron, heat treatment is a key process; not only can the matrix structure of nodular cast iron be adjusted by heat treatment to improve its performance, but also the nodulation of graphite and the distribution of alloying elements can be modified, and therefore, a much better comprehensive properties can be obtained [14,15]. It was reported that through appropriate heat treatment processes, the impact toughness of ductile iron under low-temperature conditions can be markedly enhanced, thereby avoiding the brittle fracture. This innovative approach can make much wider applications for ductile iron [16,17,18]. 

However, the specific mechanism by which heat treatment can improve the low-temperature impact toughness of ductile iron is not clarified yet. In order to make good use of the heat treatment technology, it is crucial to know the intrinsic changes occurred in ductile iron during the heat treatment process, and clarify the enhancement mechanism. Therefore, a deeper exploration into its mechanism is necessary. 

Consequently, the primary objective of this research is to conduct an in-depth investigation into the specific causes leading to performance changes in ductile iron during the heat treatment process. The aim is to discover more effective methods to promote the low-temperature impact toughness of ductile iron [19,20,21]. It is expected that the insights garnered from this research will provide valuable new perspectives for the development and application of ductile iron, leading to expanded potential for its engineering applications.

This research holds considerable value for engineering applications and contributes significance to the understanding of fundamental issues in the material science. As we move towards the future, we envision an increased application of ductile iron across various engineering fields, particularly in cold environments. In doing so, it is expected that ductile iron will play an instrumental role in propelling the advancement of human society.

## 2. Materials and Methods

**Figure 1:**
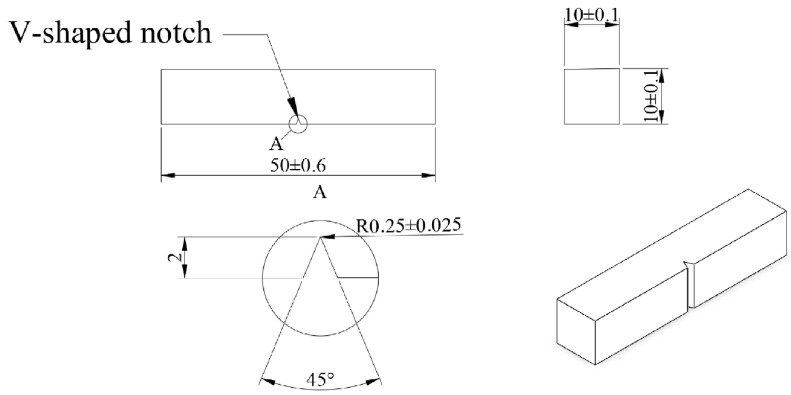
Shape and size of sample for Charpy test of impact energy.

The material used in this study was nodular cast iron with the following chemical compositions (in wt. %): 3.72 C, 2.09 Si, 0.05 Mn, 0.024 P, 0.007 S, 0.046 Mg, 0.003 La, and 0.006 Ce and Fe balance. Specimens with the size of 5 mm × 5 mm × 3 mm were cut from a piece of nodular cast iron for metallographic structure observation, EBSD test, and hardness test, and the impact energy of each sample was tested using a JB-50 Charpy tester; the shape and size of Charpy test sample was shown in Figure 1, with a V-shaped notch as marked A in Figure 1. Then, ultrasonic cleaned in anhydrous ethanol for 10–15 min prior to placing in a heat treatment furnace for heating at 930 °C for 3 h, then cooled in the furnace to room temperature.

After Charpy test of impact energy, the fracture morphology of each impact sample was observed by SEM (Zeiss, Jena, Germany) and the cleavage fracture area proportion was analyzed by software. The imager M2m (Zeiss) optical microscope was used to observe the metallographic structure, and the corrosion solution was 4% HNO_3_ in alcohol. The hardness was tested by Hvs-1000A (Huayin, China) hardness tester; the chemical composition of the matrix was analyzed by EPMA-1720 (Shimadzu, Tokyo, Japan) electron probe. The grain size and grain boundary distribution were analyzed by sigma500 (Zeiss) EBSD. The EBSD sample was prepared by mechanical grinding, polishing and electrolytic polishing with a voltage of 15 V and a polishing time of 10 s. The acceleration voltage during EBSD testing was 20 kV, the sample tilt angle was 70°, and the step size was 1 µm, and the data were analyzed by Channel 5 software (https://nano.oxinst.cn/products/ebsd/post-processing-software, Oxford Instruments, Oxford, UK).

## 3. Results and Discussion

### 3.1. Impact Energy and Fracture Morphology Analysis

**Figure 2:**
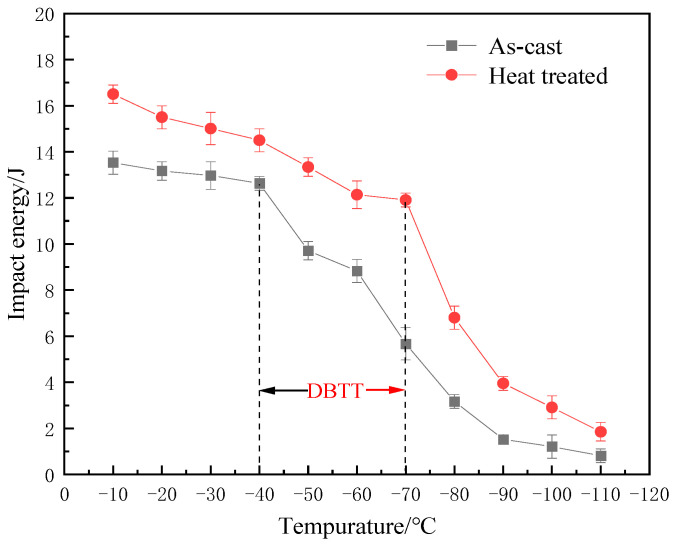
The comparison of impact energy of nodular cast iron between as-cast and heat-treated states at different temperatures.

**Figure 3:**
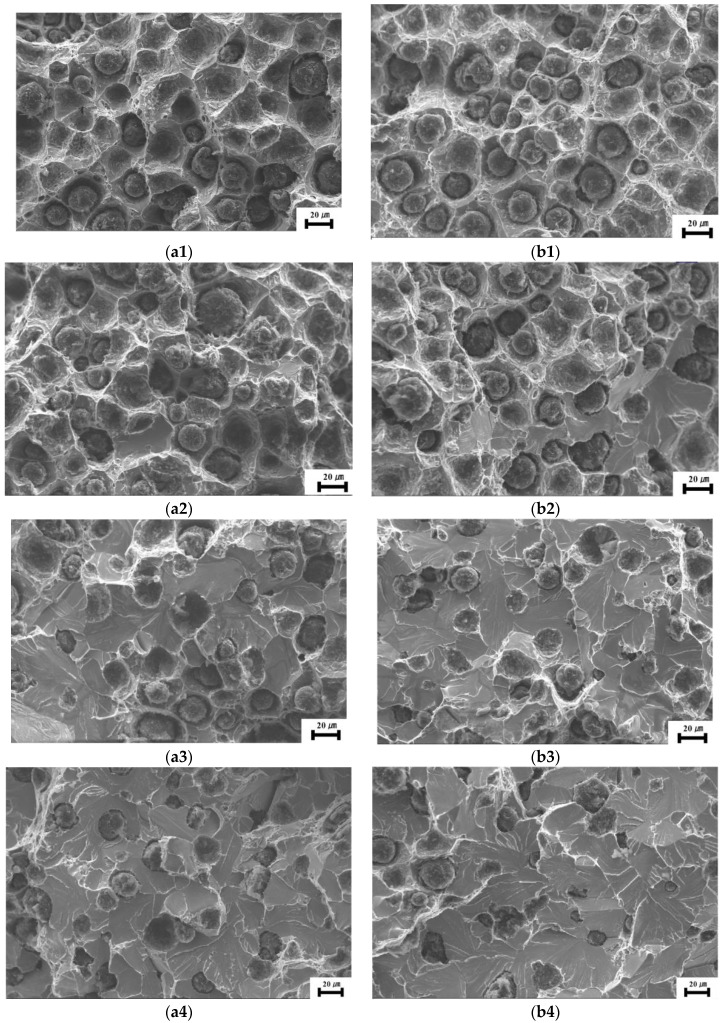

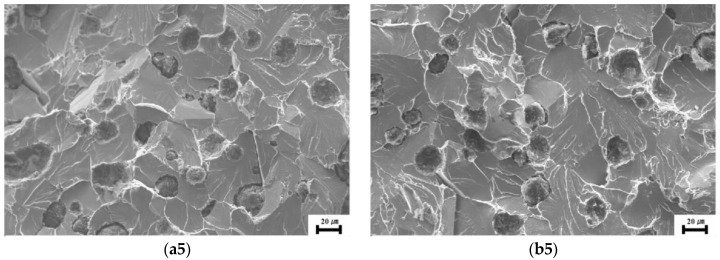
Fracture morphology. (**a**) As-cast: (**a1**) −30 °C, (**a2**) −40 °C, (**a3**) −50 °C, (**a4**) −60 °C; (**a5**) −110 °C. Heat-treated: (**b1**) −50 °C, (**b2**) −60 °C, (**b3**) −70 °C, (**b4**) −80 °C, (**b5**) −110 °C.

Impact energy is the energy necessary to fracture a standard test piece (shown in Figure 1) under an impact load, which is a similar analog of toughness, just like hardness is an analog of strength. In order to investigate the effect of heat treatment on the impact toughness of nodular cast iron, the impact energy was tested at a low-temperature range of −10 °C to −110 °C. Figure 2 and Table 1 shows the comparison of impact energy of nodular cast iron between as-cast and heat-treated states. It can be seen that though the low-temperature impact energy of the cast iron in both cases decreases with the decrease in temperature, decreasing from 13.53 J to 0.81 J for as-cast samples when the temperature decreases from −10 °C to −110 °C, while decreasing from 16.54 J to 1.85 J for heat-treated samples, it is clear that the impact energy in heat-treated state is obviously higher than that in as-cast state at any temperature, which means that the impact toughness of nodular cast iron is enhanced by heat treatment. And it needs to be noted that the impact energy decreases sharply at temperatures lower than −40 °C for as-cast nodular cast iron, while it keeps decreasing gently until −70 °C for heat-treated nodular cast iron, and turns to decrease sharply at temperatures lower than −70 °C, which demonstrates that the ductile-to-brittle transition temperature (DBTT) can be decreased from about −40 °C to −70 °C by heat treatment, as the dotted lines show (in Figure 2). The decrease in the ductile-to-brittle transition temperature makes the impact energy difference between as-cast and heat-treated states much bigger at a temperature range of −50 °C~−70 °C, which is attributed to the brittle fracture for as-cast nodular cast iron turning to be ductile fracture by heat treatment at this temperature range. Therefore, heat treatment makes nodular cast iron suitable for application in environments subjected to much lower temperatures. Also, it can be seen that the number of micro cracks that occurred in as-cast iron is higher than that in heat-treated cast iron, as shown in Figure 3(a5,b5).

In order to further understand the effect of heat treatment on the impact toughness of nodular cast iron, the corresponding fracture morphology of each specimen was observed by SEM after each impact test, and the cleavage fracture area proportion was analyzed by software. Figure 3 presents the fracture morphology of nodular cast iron in both as-cast and heat-treated states at different temperatures, and the comparison of cleavage fracture area proportion of nodular cast iron in both cases is also shown in Table 1. It can be clearly seen that the cleavage fracture area proportion increases with the decrease in temperature in both cases, increasing from 5% to 83% for as-cast samples when temperature decreasing from −10 °C to −110 °C, while increasing from 4% to 85% for heat-treated samples; it is clear that the cleavage fracture area proportion in heat-treated state is obviously less than that in as-cast state at any temperature, which further confirms that the impact toughness of nodular cast iron is improved by heat treatment. And it can also be seen that the difference in cleavage fracture area proportion between as-cast and heat-treated states is much bigger at a temperature range of −50 °C~−70 °C due to the decrease in the ductile brittle transition temperature by heat treatment, which is in good agreement with the difference in the impact energy in both cases.

### 3.2. Metallographic Microstructure Analyses

It is known that heat treatment has an important effect on the size, quantity, and morphology of nodules in nodular cast iron, because the nodules can act as the stress concentration point, prevent crack propagation, and provide certain dislocation absorption capacity. However, nodules of too large a size may decrease the strength of the cast iron, and though an increase in the quantity of nodules can improve the low-temperature impact toughness of nodular cast iron, since they can effectively prevent crack propagation and provide a dislocation slip path that absorbs the impact energy, too many nodules can decrease the strength and hardness of the cast iron. Generally, a suitable size and amount of nodules can improve the low-temperature impact toughness of nodular cast iron, because they can provide an effective dislocation slip path and prevent crack propagation. The irregular or clustered spheroidal morphology may lead to stress concentration and crack propagation, thereby reducing low-temperature impact toughness.

**Figure 4:**
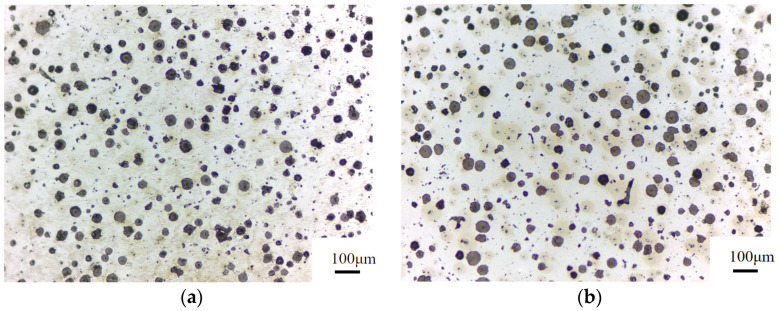
Metallographic structure of nodular cast iron. (**a**) As-cast; (**b**) heat treated.

To investigate the effect of heat treatment on the morphology, size, and quantity of graphite, and the microstructure of matrix of nodular cast iron, a comparison was made between the metallographic structure of the as-cast and heat-treated samples. Figure 4a shows the microstructure of the as-cast sample, and Figure 4b shows the microstructure of the heat-treated sample. 

In order to have a reliable comparison of the quantity and size of spherical graphite before and after heat treatment, quantitative indicators were obtained in the range of screen, as can be seen that the quantity of spherical graphite is 211 and 219, respectively; and the ratio of spherical graphite is 20% and 19%, respectively, for the as-cast and heat-treated samples, and it can be seen that the size and quantity of spherical graphite remains almost the same in both cases. Thus it can be concluded that the microstructure of matrix, the morphology, size, and quantity of spherical graphite basically remains unchanged after heat treatment. Thus, it is inferred that the increase in low-temperature impact toughness of the heat-treated sample does not result from the change in the microstructure of the nodular cast iron.

### 3.3. Vickers Hardness Analysis

To clarify the enhancement mechanism of low-temperature impact toughness of nodular cast iron by heat treatment, the effect of heat treatment on the hardness of nodular cast iron was investigated. Ten Vickers hardness tests were conducted on the as-cast and heat-treated samples; the hardness value is shown in Table 2. It can be seen that the Vickers hardness after heat treatment is relatively stable and uniform. Although the hardness decreases slightly after heat treatment, the decrease ratio is much lower than the increase ratio of impact energy as shown in Table 2. Thus, it can be inferred that the main reason for the increase in impact toughness by heat treatment is not resulted from the decrease in hardness.

### 3.4. Micro-Area Composition Analysis

Since heat treatment may affect the distribution of Si, Mn, and C elements in nodular cast iron, it thus brings about an impact on the properties. Appropriate Si content can improve the low-temperature impact toughness of ductile iron because Si can promote the formation and stability of nodular graphite and reduce the formation of brittle phase (such as hard bainite), thereby increasing the toughness of the cast iron. Meanwhile, appropriate Mn content can also improve the low-temperature impact toughness of ductile iron because Mn can promote the formation of stable carbides, preventing the formation of brittle phases (such as acicular pearlite).

**Figure 5:**
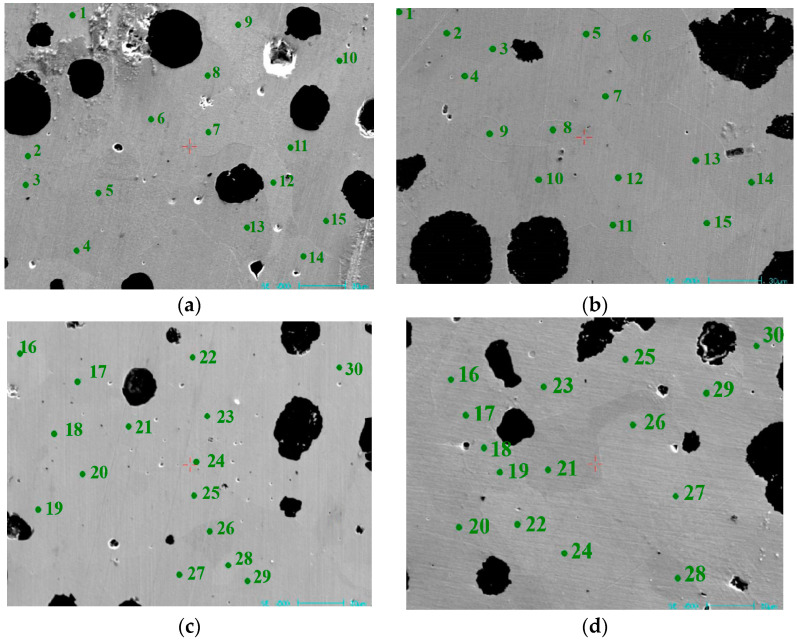
Electron probe test points. (**a**,**c**) As-cast; (**b**,**d**) heat treated.

Due to the possible exchange of elements between graphite and matrix during heat treatment process of nodular cast iron, and especially, Si, Mn, and C have significant impact on its performances, the Si, Mn, and C content of the as-cast and heat-treated samples was analyzed using an electron probe. A total of 30 points were tested for each sample, as shown in Figure 5. The corresponding contents of each point in as-cast and heat-treated states are shown in Table 3. It can be seen that although the content of Si, Mn, and C varies slightly after heat treatment, the difference can be almost ignored. Therefore, it can be speculated that the effect of heat treatment on the exchange of elements in the matrix is not the main reason for the significant improvement in the impact toughness of nodular cast iron.

### 3.5. Grain Size and Grain Boundary Distribution Analysis

**Figure 6:**
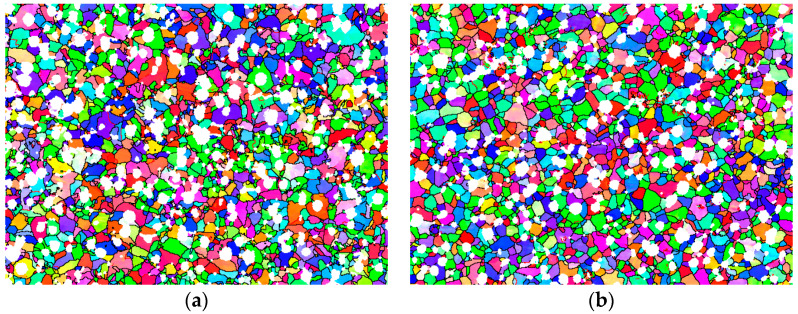
Grain size and orientation. (**a**) As-cast; (**b**) heat treated.

The smaller grain size increases the grain boundary area and makes the crack propagation path more complex, thus improving the anti-crack propagation ability of the material, which can improve the toughness of ductile iron. Therefore, we compared the grain size and misorientations of the as-cast and heat-treated samples. Figure 6 shows the grain size and orientation maps of two kinds of samples, with different colors representing different misorientations between grains. The white represents graphite, the other colors represent the ferrite grains with different crystal orientations, and different colors correspond to different orientations.

**Figure 7:**
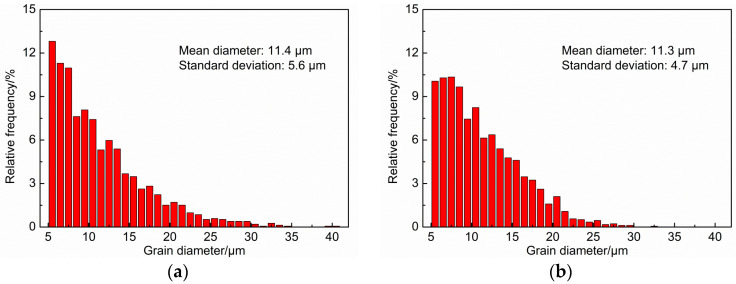
Distribution of grain size. (**a**) As-cast; (**b**) heat treated.

According to Figure 7, the grain size and its distribution of the two groups of samples were compared, as shown in Figure 7. Among them, Figure 7a is the distribution map of the grain size for the as-cast sample, and Figure 7b is the distribution of the grain size for the heat-treated sample. From the figure, it can be seen that the average diameter is 11.4 µm and 11.3 µm for the as-cast and heat-treated samples, respectively. In other words, the average grain size of the two groups of samples is basically the same, though the proportion of fine grains decreases slightly, and the uniformity of grain size increases slightly after heat treatment.

Grain boundaries play an important role in determining material properties, especially low-temperature impact toughness, which can produce plastic deformation under impact loading, so as to absorb the impact energy, and prevent crack propagation, thus improving the toughness of the material. Therefore, by adjusting and controlling the grain boundaries of nodular cast iron, the low-temperature impact toughness can be effectively improved. 

**Figure 8:**
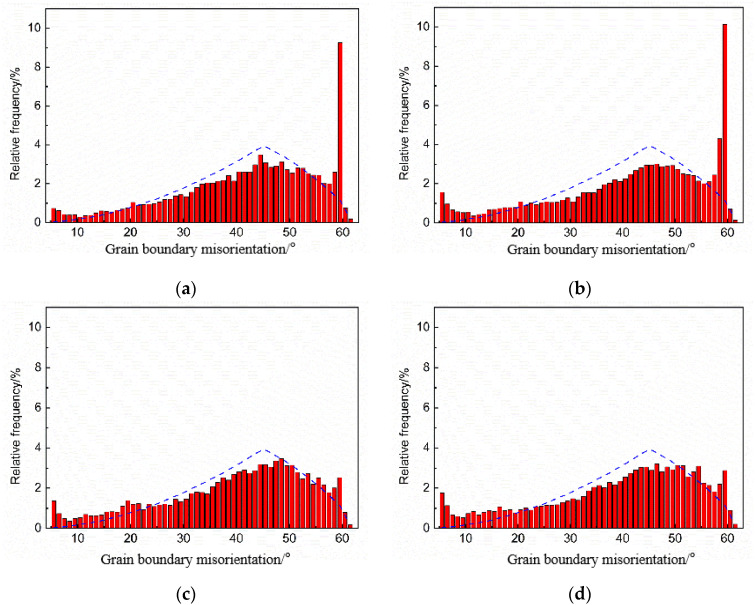
Distribution of grain boundaries (59°~60°). (**a**,**b**) As-cast; (**c**,**d**) heat treated.

To investigate the effect of heat treatment on the grain boundaries of nodular cast iron, the distribution of grain boundaries of the as-cast and heat-treated samples was analyzed and compared. Figure 8a,b show the distribution of grain boundaries of the as-cast sample, Figure 8c,d show the distribution of grain boundaries of the heat-treated sample. From the figure, it can be seen that the proportion of HAGB between 59° and 60° in the as-cast sample exceeds 10%, while the proportion of those decreases dramatically to less than 3% after heat treatment, i.e., more than 70% less than that of the as-cast sample. 

Considering the influence of heat treatment on all the above aspects, it can be seen that the maximum difference between as-cast and heat-treated nodular cast iron is the proportion of HAGB between 59° and 60°. Therefore, it is reasonable to conclude that the main reason for improving the low-temperature impact toughness of nodular cast iron by heat treatment is the significant decrease in HAGB between 59° and 60°. In other words, the decrease in HAGB between 59° and 60° brings about better low-temperature impact toughness. 

## 4. Discussion

Impact energy and fracture morphology clearly illustrate that the low-temperature impact toughness of nodular cast iron can be significantly enhanced by heat treatment, and the systematic analysis and comparison of the microstructure, hardness, composition, and other characteristics of as-cast and heat-treated nodular cast iron confirmed that heat treatment has little effect on the morphology and size of the graphite, ignores the effect on the grain size, morphology and distribution of ferritic matrix, and has little effect on the hardness and exchange of elements, while heat treatment can bring about a significant decrease in high-angle grain boundaries (HAGB) between 59° and 60°, decreasing from 10% to 3%. Therefore, it is concluded that the significant enhancement of low-temperature impact toughness of nodular cast iron by heat treatment may result from the decrease in HAGB between 59° and 60°.

How can the decrease in HAGB between 59° and 60° enhance the impact toughness of nodular cast iron at low temperatures? The possible reason is that during plastic deformation, the dislocations can move along the slip plane within the grains upon subjecting the impact load, thus forming dislocation accumulation at the intersection of the slip planes, resulting in stress concentration and the formation of initial cracks. However, due to the different orientation of each grain, the initial crack propagation to the grain boundary will be hindered by the grain boundary, causing significant stress concentration near the grain boundary and forming a crack source. When these cleavage cracks propagate forward and approach each other, the connected metal is quickly torn apart due to high stress, forming fractures that originate from grain boundaries [22,23,24]. 

The characteristic of high-angle grain boundaries (HAGB) between 59° and 60° is high lattice mismatch, and there is a significant irregularity in the atomic arrangement between grains, which hinders the movement of dislocations in the crystal and causes initial cracks to concentrate at the grain boundaries to form crack sources. Therefore, the reduction in high-angle grain boundaries (HAGB) can significantly improve the low-temperature impact toughness of nodular cast iron.

## 5. Conclusions

The impact energy of nodular cast iron at a low-temperature range of −10 °C to −80 °C and the analysis of corresponding fracture morphology confirmed that heat treatment could enhance the impact toughness of the nodular cast iron, this research focused on exploring the enhancement mechanism of heat treatment on the low-temperature impact toughness of nodular cast iron; the following main conclusions can be drawn:

(1) Heat treatment has little effect on the morphology and size of graphite in nodular cast iron, and it also has little effect on the grain size, morphology, and distribution of the ferritic matrix.
(2) Heat treatment has no significant effect on the hardness and exchange of elements.
(3) Heat treatment brings about significant decrease in the proportion of high-angle grain boundaries (HAGB) between 59° and 60°, decreasing from 10% to 3%.
(4) The significant enhancement of low-temperature impact toughness of nodular cast iron may result from the significant decrease in the proportion of HAGB between 59° and 60° by heat treatment.
(5) This study may provide novel ideas for optimizing the heat treatment process of nodular cast iron.

## Figures and Tables

**Table 1:** Comparison of impact energy and cleavage fracture area proportion of nodular cast iron between as-cast and heat-treated states.

Temperature/°C	Impact Energy/J		Cleavage Fracture Area Proportion/%	
	As-Cast	Heat Treated	As-Cast	Heat Treated
−10	13.53	16.54	5	4
−20	13.17	15.52	7	6
−30	12.97	15.01	8	7
−40	12.63	14.50	11	10
−50	9.71	13.34	53	11
−60	8.83	12.14	67	19
−70	5.67	11.91	73	67
−80	3.17	6.80	77	79
−90	1.52	3.95	80	73
−100	1.21	2.91	81	78
−110	0.81	1.85	83	85

**Table 2:** Vickers hardness of nodular cast iron in as-cast and heat-treated states.

Point	Vickers Hardness/HV	
	As-Cast	Heat Treated
1	143	127
2	141	139
3	140	135
4	144	131
5	136	142
6	147	130
7	145	128
8	140	135
9	138	128
10	141	133
Average	142	133
Standard deviation	3.3	5.0

**Table 3:** Comparison of Si, Mn, and C content between as-cast and heat-treated nodular cast iron.

Point	Si		Mn		C	
	As-Cast	Heat Treated	As-Cast	Heat Treated	As-Cast	Heat Treated
1	2.232	2.661	0.032	0.042	2.053	2.239
2	2.566	2.535	0.003	0.003	2.553	1.191
3	2.306	2.209	0.071	0.061	2.083	1.563
4	2.203	2.458	0.081	0.021	1.732	2.359
5	2.613	2.588	0.032	0.031	1.977	2.018
6	2.531	2.624	0.009	0.003	3.032	0.698
7	2.144	2.543	0.064	0.005	2.449	2.511
8	1.834	2.687	0.114	0.049	1.182	2.053
9	1.752	2.111	0.116	0.006	1.383	1.092
10	2.109	1.953	0.055	0.055	1.157	1.289
11	2.693	2.391	0.022	0.037	1.683	1.322
12	2.313	2.234	0.075	0.009	1.599	0.907
13	2.624	2.394	0.011	0.048	2.721	1.668
14	2.381	1.478	0.082	0.012	2.532	0.176
15	2.036	2.469	0.059	0.004	0.983	0.916
16	1.696	2.106	0.031	0.041	1.272	5.173
17	2.384	2.314	0.005	0.003	1.916	1.871
18	2.233	1.955	0.071	0.061	1.128	1.329
19	2.478	2.016	0.079	0.021	2.284	1.787
20	2.566	2.293	0.032	0.029	1.892	5.019
21	2.275	2.413	0.009	0.003	1.908	1.994
22	2.641	2.113	0.064	0.002	2.655	1.932
23	2.252	2.132	0.112	0.048	2.012	5.217
24	2.465	2.501	0.115	0.007	1.817	1.071
25	2.133	2.518	0.054	0.049	1.322	3.833
26	2.241	2.044	0.021	0.036	1.499	1.631
27	2.193	1.952	0.074	0.009	2.066	1.289
28	2.358	2.117	0.011	0.047	1.998	1.663
29	2.368	2.194	0.081	0.024	2.431	5.592
30	2.527	2.438	0.058	0.008	1.829	1.721
Average value	2.305	2.281	0.0548	0.026	1.905	2.104
Maximum value	2.693	2.687	0.116	0.061	3.032	5.592
Minimum value	1.696	1.478	0.003	0.002	0.983	0.176
Standard deviation	0.254	0.272	0.0351	0.020	0.526	1.417

## Data Availability

The data that support the findings of this study are available from the corresponding author.
